# Genome Erosion in a Nitrogen-Fixing Vertically Transmitted Endosymbiotic Multicellular Cyanobacterium

**DOI:** 10.1371/journal.pone.0011486

**Published:** 2010-07-08

**Authors:** Liang Ran, John Larsson, Theoden Vigil-Stenman, Johan A. A. Nylander, Karolina Ininbergs, Wei-Wen Zheng, Alla Lapidus, Stephen Lowry, Robert Haselkorn, Birgitta Bergman

**Affiliations:** 1 Department of Botany, Stockholm University, Stockholm, Sweden; 2 Biotechnology Research Center, Fujian Agriculture and Forestry University, Fuzhou, China; 3 Department of Energy Joint Genome Institute, Walnut Creek, California, United States of America; 4 Department of Molecular Genetics and Cell Biology, University of Chicago, Chicago, Illinois, United States of America; University of Hyderabad, India

## Abstract

**Background:**

An ancient cyanobacterial incorporation into a eukaryotic organism led to the evolution of plastids (chloroplasts) and subsequently to the origin of the plant kingdom. The underlying mechanism and the identities of the partners in this monophyletic event remain elusive.

**Methodology/Principal Findings:**

To shed light on this evolutionary process, we sequenced the genome of a cyanobacterium residing extracellularly in an endosymbiosis with a plant, the water-fern *Azolla filiculoides* Lam. This symbiosis was selected as it has characters which make it unique among extant cyanobacterial plant symbioses: the cyanobacterium lacks autonomous growth and is vertically transmitted between plant generations. Our results reveal features of evolutionary significance. The genome is in an eroding state, evidenced by a large proportion of pseudogenes (31.2%) and a high frequency of transposable elements (∼600) scattered throughout the genome. Pseudogenization is found in genes such as the replication initiator *dnaA* and DNA repair genes, considered essential to free-living cyanobacteria. For some functional categories of genes pseudogenes are more prevalent than functional genes. Loss of function is apparent even within the ‘core’ gene categories of bacteria, such as genes involved in glycolysis and nutrient uptake. In contrast, serving as a critical source of nitrogen for the host, genes related to metabolic processes such as cell differentiation and nitrogen-fixation are well preserved.

**Conclusions/Significance:**

This is the first finding of genome degradation in a plant symbiont and phenotypically complex cyanobacterium and one of only a few extracellular endosymbionts described showing signs of reductive genome evolution. Our findings suggest an ongoing selective streamlining of this cyanobacterial genome which has resulted in an organism devoted to nitrogen fixation and devoid of autonomous growth. The cyanobacterial symbiont of *Azolla* can thus be considered at the initial phase of a transition from free-living organism to a nitrogen-fixing plant entity, a transition process which may mimic what drove the evolution of chloroplasts from a cyanobacterial ancestor.

## Introduction

Photosynthetic plastids were introduced about two billion years ago in a monophyletic endosymbiotic event that led to the genesis of a successful new kingdom, that of embryophytic algae and eventually land plants [1], [2]. The host gained an enormous fitness advantage, namely a mechanism for capturing light-energy to fix carbon dioxide (photosynthesis). The evolutionary success of this event is today manifested in the more than 250,000 species of flowering plants, second in number only to insects. The emergence of oxygenic photosynthesis gradually re-shaped the bio- and atmosphere of the globe [3]. Inasmuch as this event occurred nearly two billion years ago it is understandable that we have scant knowledge of the evolutionary process and the exact nature of the organisms involved in chloroplast genesis. It is however clear that the endosymbiont was a cyanobacterium (likely of the same type as the cyanobacteria seen in plant symbioses today: i.e. a filamentous, heterocystous cyanobacterium [4]) that on integrating with the host was transformed into a new organelle, vertically maintained between host generations. This ‘ancient cyanobacterium’ has today one of the smallest genomes known (150-200 kbp), due to extensive loss and transfer of genes to the host nucleus [5]. The obligate symbiosis subsequently led to a shrinking of the symbiont genome, streamlining its functions towards photosynthesis and an obligate life style as organelles in all plants.

**Figure 1:**
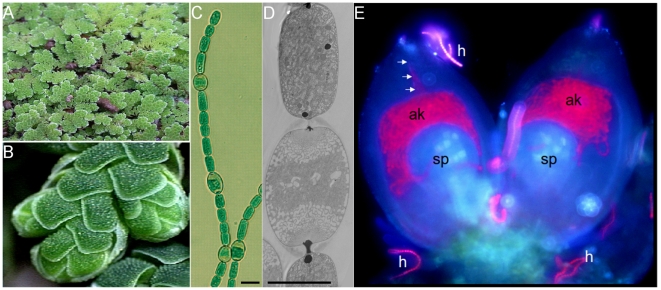
The partners in the *Azolla* symbiosis. A) Fronds of the *Azolla filiculoides* Lam. plant. B) Close up of an *Azolla* branch showing the apex and the alternating ‘stacked’ dorsal leaves, each containing a cavity in which the cyanobiont (NoAz) filaments reside. C) Light micrograph of the cyanobiont. The larger cells in the vegetative filaments represent the nitrogen-fixing heterocysts. Scale bar  = 5 µm. D) Transmission electron micrograph of the cyanobiont. Note the thicker cell-walls and the electron dense polar nodules of the heterocyst (middle cell) at the interface to flanking vegetative cells, which function as combined N storage structures (cyanophycin). Scale bar  = 5 µm E) A snap-shot in the vertical transmission process of the cyanobiont between *Azolla* plant generations, using fluorescence microscopy. Pairs of megasporocarps (blue) develop at the underside of the cyanobacterial colonized *Azolla* leaves. Filaments of the motile cyanobacterial cell stage (red), the hormogonia (h), are attracted to the sporocarps, gather at the base and subsequently move towards the tip, before entering the sporocarps via channels (white arrows). Once inside the sporocarp the hormogonia differentiate into individual thick walled resting spores (or akinetes; ak), seen as the intensively red fluorescing small inoculum on top of the megaspores (sp). For details see [15].

Some contemporary cyanobacteria are symbiotically highly competent and are able to colonize a range of plant lineages [6]. In contrast to the ‘primary’ endosymbionts, the chloroplasts, these represent a ‘second round’ of cyanobacterial invasions. In these, the cyanobacterium has taken on a new physiological role, i.e. to fulfill the full nitrogen demands of the host via their enhanced nitrogen-fixing capacity [7]. A striking feature of the cyanobacterial symbionts (cyanobionts) of plants is their complex phenotypic appearance, all being filamentous and capable of differentiating various cell types elicited by external cues. The process of genome reduction, described extensively for *intracellular* bacterial symbionts and parasites of insects [8]–[11], has recently been shown to also influence the genomes of *extracellular* symbiotic bacteria [12], [13]. We thus hypothesized that, given the right conditions, genome reduction may also act on cyanobacteria in symbiosis with plants. We also hypothesize that such evolutionary mechanisms, when specifically acting on cyanobacteria, may resemble what governed the evolution of chloroplasts from a cyanobacterial ancestor. By understanding these mechanisms we may get insights into the seminal process that led to the evolution of the eukaryotic plant cell and the plant kingdom. To test our hypothesis, we selected the nitrogen-fixing symbiont of the small heterosporous water fern *Azolla filiculoides* Lam [14] as a model system. *Azolla* is a fast-growing aquatic fern (Figure 1A–B), which are colonized by a filamentous nitrogen-fixing cyanobiont (Figure 1C–D). This cyanobiont resides as restricted populations in specialized ‘cavities’ in each individual dorsal plant leaf. The nitrogen fixed is released and transferred to the host plant, via an unidentified mechanism. The *Azolla* endosymbiosis displays some evolutionary intriguing features. First, the host is able to maintain a small proportion of the cyanobiont population as an ‘inoculum’ between plant generations [14], [15]. This is accomplished through a complex and unique process using the *Azolla* reproductive organ, the sporocarp (predecessors to plant seeds), as transfer vehicle (Figure 1E). This process relies on the capacity of the cyanobiont to differentiate motile filamentous hormogonia, which are attracted to and enter the sporocarp through a narrow pore. On entering the sporocarp the hormogonia differentiate into a resting stage (spores/akinetes), in which they remain dormant (extracellularly) until the plant germinates [15]. Secondly, the cyanobacterial partner seems to have lost (at least part of) its autonomy as it can not grow outside the plant [15], [16]. Such features suggest a long-lasting co-evolution between the partners, potentially extending back for as long as 140 million years (oldest fossil records of *Azolla* [17]). We here report on the genomic properties of this cyanobiont, suggest reasons for its obligate host-dependency and provide evidence for a progressing streamlining of its genome for nitrogen fixation. The significance of our findings for plant and symbiont evolution are discussed.

## Results and Discussion

**Figure 2:**
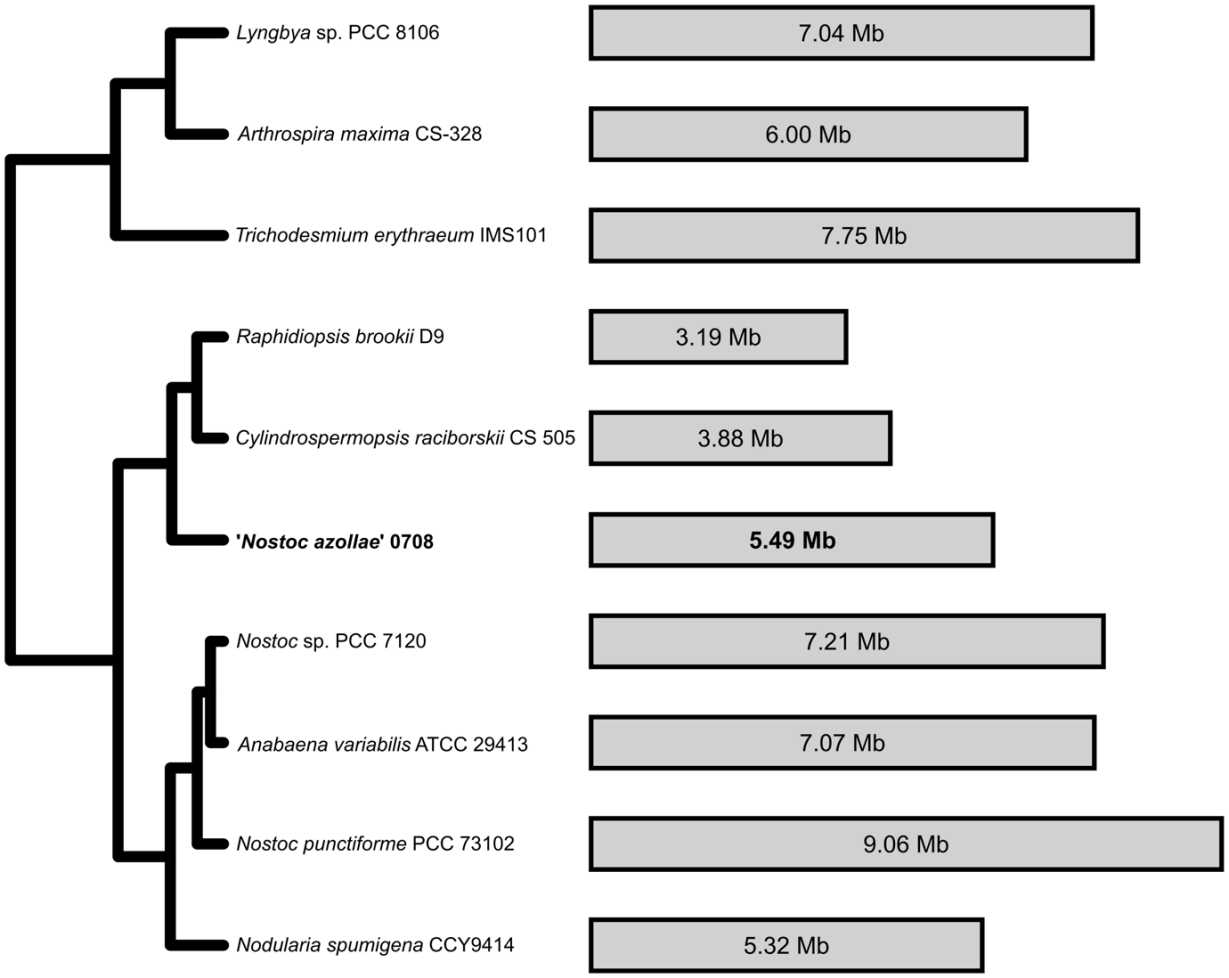
Phylogenetic tree and genome sizes for ten filamentous cyanobacterial species. The closest relatives to ‘*Nostoc azollae*’ 0708 are *Raphidiopsis brookii* D9 and *Cylindrospermopsis raciborskii* CS 505, the two multicellular cyanobacteria with the smallest known genomes. The tree is a subclade from a maximum likelihood analysis of all cyanobacterial genomes available from NCBI and IMG/ER (see Material and Methods).

The cyanobiont of the water-fern *A. filiculoides* (‘*Nostoc azollae*’ 0708, hereafter referred to as NoAz) is a filamentous diazotrophic cyanobacterium of Section IV [18]. It is able to differentiate cells devoted to nitrogen fixation (heterocysts, Figure 1C–D), motile small celled hormogonia, used in the unique vertical transmission process, and resting akinetes (Figure 1E). Unexpectedly, our phylogenetic analysis does not place NoAz together with the assumed closely related *Nostoc/Anabaena/Nodularia* species (Figure 2). Instead, NoAz is sistergroup to two recently sequenced cyanobacteria with small genomes, *Raphidiopsis brokii* D9 and *Cylindrospermopsis raciborskii* CS-505 [19].

### The genome

**Figure 3:**
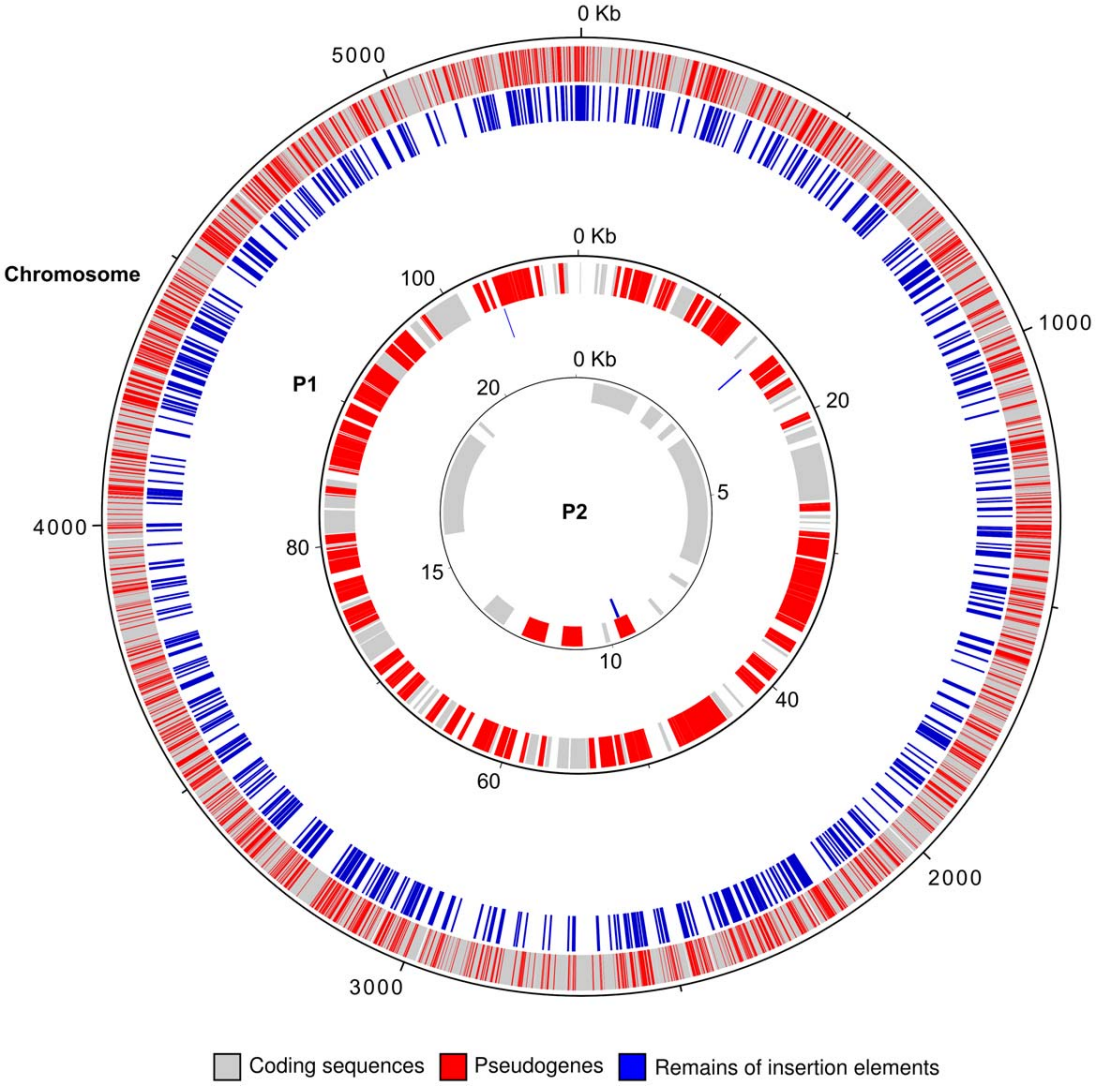
Map of the main chromosome, and plasmids (P1, P2) of the ‘*Nostoc azollae*’ 0708 genome. The distribution of pseudogenes (red) and remains of insertion elements (blue) are indicated. Predicted genes are indicated by grey color. The highest level of gene erosion (number of pseudogenes:number of predicted genes) is found in the plasmid P1. Note that the occurrence of insertion elements appears to be correlated with the distribution of pseudogenes. The P1 and P2 plasmids only contain two and one remains of insertion elements, respectively.

The genome sequence of the cyanobiont consists of one chromosome and two plasmids (Genbank accession numbers: CP002059, CP002060, CP002061) (Figure 3) encompassing a total of 5,486,145 bp with a relatively low G+C content of 38.3% (Table 1). It contains four rRNA clusters and 44 species of tRNA, representing the full set of amino acids. Of the 5,357 coding sequences (CDS) predicted in the NoAz genome, 3,668 have intact open reading frames while the rest are pseudogenes. Intact genes together comprise 52% of the genome, a coding fraction which is lower than in any other cyanobacterial genome sequenced. Additionally, the number of intact CDS is among the lowest in the filamentous cyanobacteria sequenced to date.

**Table 1:** Overview of genome features in the cyanobiont (‘*Nostoc azollae*’ 0708) of the water fern *Azolla filiculoides* Lam.

Feature	Chlp*	**NoAz**	Noss+	Nosp+
Symbiotic competence	Obligate	**Obligate**	None	Facultative
Genome size (bp)	154,478	**5,486,145**	7,211,789	9,059,191
Plasmids	0	**2**	6	5
Coding nucleotide proportion %	51	**52**	82	77
GC content %	36	**38**	41	41
Genes, total number	129	**5413**	6222	6791
Coding sequences	85	**3668**	6,130	6,690
Pseudogenes (%)	0	**1689 (31.2)**	0	0
rRNA	7	**12**	12	12
tRNA	37	**44**	70	88

For comparative purposes the genomes of a chloroplast (Arabidopsis) and genomes of two related cyanobacteria (Section IV), one being a facultative plant symbiont and the other a free-living species, are given. Chlp  =  Chloroplast of *Arabidopsis thaliana*, NoAz  =  ‘*Nostoc azollae*’ 0708, Noss  =  *Nostoc* sp. PCC 7120, Nosp  =  *Nostoc punctiforme* PCC 73102.

* Data from NCBI database (http://www.ncbi.nlm.nih.gov/).

+ Data from IMG database (http://img.jgi.doe.gov/).

### Pseudogenes and insertion sequences

**Figure 4:**
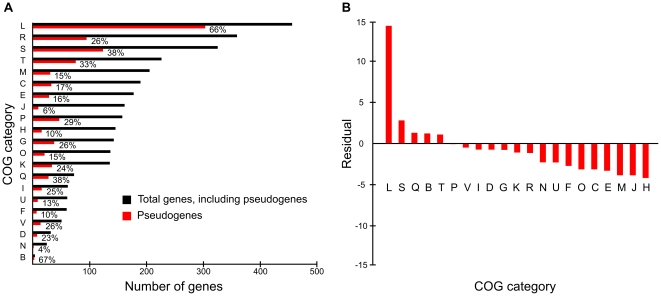
Classification of genes in COG functional categories. A) Distribution of genes and pseudogenes in functional categories. Percentages signify the amount of pseudogenes in each category. A Pearson's Chi-squared test (see Materials and Methods) shows that the distribution of pseudogenes within COG categories is non-random. B) Residuals from the Pearson's Chi-squared test. Large positive values indicates a stronger overrepresentation of pseudogenes while large negative values indicate stronger underrepresentation. (B)  =  Chromatin structure and dynamics, (C)  =  Energy production and conversion, (D)  =  Cell cycle control, cell division, chromosome partitioning, (E)  =  Amino acid transport and metabolism, (F)  =  Nucleotide transport and metabolism, (G)  =  Carbohydrate transport and metabolism, (H)  =  Coenzyme transport and metabolism, (I)  =  Lipid transport and metabolism, (J)  =  Translation, ribosomal structure and biogenesis, (K)  =  Transcription, (L)  =  Replication, recombination and repair, (M)  =  Cell wall/membrane/envelope biogenesis, (N)  =  Cell motility, (O)  =  Posttranslational modification, protein turnover, chaperones, (P)  =  Inorganic ion transport and metabolism, (Q)  =  Secondary metabolites biosynthesis, transport and catabolism, (R)  =  General function prediction only, (S)  =  Function unknown, (T)  =  Signal transduction mechanisms, (U)  =  Intracellular trafficking, secretion, and vesicular transport, (V)  =  Defense mechanisms.

A notable feature of the NoAz genome is the large proportion (31.2%) of pseudogenes (see Methods). This may have dramatic consequences for the genome structure and the function of the *Azolla* endosymbiont. Pseudogenes are present scattered throughout the NoAz genome (Figure 3). The large proportion indicates that the genome is in a state of degradation [8]. The highest level of gene erosion, analyzed as number of pseudogenes:number of predicted genes, is found in the plasmid P1 (63:51), followed by the chromosome (1623:3606), and plasmid P2 (3∶11). Pseudogenes are known to accumulate in endosymbiotic organisms residing in a sheltered environment with low exposure to genetic parasites, such as horizontally transferred DNA, bacteriophages and transposons, since the benefits of genomic deletions are removed [9]. In spite of being extracellular, the cyanobiont population in the *Azolla* leaf cavities is contained in a highly sheltered environment. Classification of the NoAz pseudogenes into COG functional categories illustrates their proliferation within all genomic functions (Figure 4A). A Pearson's Chi-squared test (see methods) shows that the distribution of pseudogenes within COG categories is non-random (p-value  =  0.0004998). A significant overrepresentation of pseudogenes is particularly evident in the category Replication, recombination and repair (L) but is also displayed in the categories Secondary metabolites biosynthesis and metabolism (Q), Chromatin structure and dynamics (B), Signal transduction mechanisms (T) and Function unknown (S) (Figure 4B). Conversely, functional categories with a relative underrepresentation of pseudogenes include the Coenzyme transport and metabolism (H), Translation (J) and Cell wall/membrane/envelope biogenesis (M) categories.

**Figure 5:**
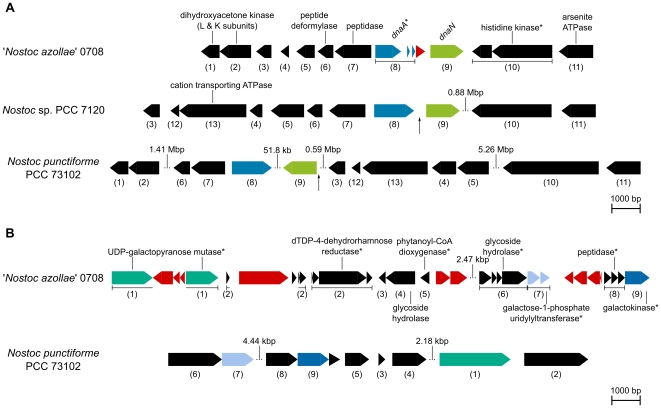
Examples of gene fragmentation in ‘*Nostoc azollae*’ 0708 (NoAz) compared to other cyanobacteria. *Nostoc punctiforme* PCC 73102 (Nosp) and *Nostoc* sp. PCC 7120 (Noss). Best reciprocal BLAST hits between genomes are indicated for each image subset by numbers in parenthesis below genes. Transposases are seen in red. Pseudogenes are indicated by the * suffix. Gaps in the sequence are indicated by three dots and the length of the omitted sequence. A) The *dnaA* region. Vertical black arrows indicate *oriC* regions predicted by Ori-Finder (see Materials and Methods). Note the fragmentation of the *dnaA* gene and the putative transposase between *dnaA* and *dnaN* in NoAz. Although large genomic parts appear to have been lost from the NoAz genome, the organization of several genes in the different species is conserved. B) A cluster of genes involved in galactose/polysaccharide metabolism. This gene cluster is not present in any other cyanobacterial genome in the IMG database. Note that genes in NoAz are heavily fragmented in comparison to Nosp and that the gene organization is rearranged. Transposases are present between ORFs and also within the UDP-galactopyranose mutase gene in NoAz. The genes encoding transposases are also fragmented.

An unexpected and notable pseudogene in the NoAz genome is the DNA replication initiator, *dnaA* (L category) (Figure 5A), which suggests severe problems related to cell multiplication. As *dnaA* is also absent in an obligate insect endosymbiont [20] a pseudogenization of *dnaA* in NoAz may indicate a selective genome reduction related to a need of the host plant to restrain DNA replication and growth of the endosymbiont. However, the cyanobiont still divides, albeit slowly, and is able to differentiate the various cell types required to maintain its role as a perpetual nitrogen-fixing endosymbiont. As a *dnaA* mutant of the unicellular cyanobacterium *Synechocystis* sp. PCC 6803 exhibits wild-type growth characteristics [21], alternative DnaA-independent replication mechanism(s) [22] may also exist in NoAz. Pseudogenization has also affected the plasmid encoded DNA replication genes *dnaX, dnaN, holB,* and *ssb*, while intact counterparts exist on the chromosome. In addition, the DNA repair genes *recD* and *alkA* are pseudogenes, as is one of two copies of the DNA helicase gene *recQ*. The loss of function in the DNA repair category may have promoted the accumulation and spread of pseudogenes in NoAz genomic functions, including in the DNA replication process. Additionally, more than 600 distinct sites in the NoAz genome contain remains of insertion sequences (ISs) of which only two and one are located on the plasmids P1 and P2, respectively (Figure 3). ISs are 700–3000 nt long mobile DNA sequences, containing a transposase encoding gene flanked by inverted repeats [23]. The transposase facilitates the excision and integration of ISs in the genome. However, automated annotation identifies only 67 “transposase” ORFs. Of these, only three are longer than 200 amino acids, indicating that most of the transposases in NoAz are no longer functional. This is not surprising, as IS elements are not critical to the organism and therefore prone to pseudogenization. IS elements with a fragmented transposase can still be mobile, however. As insertion elements commonly exist in multiple locations within the genome, a remaining copy with a functional transposase can compensate the loss of function in other insertion elements. Insertion elements proliferate in genomes of endosymbiotic microbes and particularly in those that have recently evolved a host-restricted lifestyle [10], but are lacking in genomes of truly ancient endosymbionts [24], including chloroplasts. The insertion sequences in NoAz, are found interrupting ORFs of a number of genes, and genes in proximity to insertion element remains are often fragmented (Figure 3, 5A, B). Together these findings argue for a disruption of vital genomic functions in the *Azolla* cyanobiont, most likely underpinned by the lowered evolutionary pressure in the cryptic *Azolla* leaf cavities.

### Functional comparison to phylogenetically related cyanobacteria

Although NoAz groups phylogenetically with *C. raciborskii* CS-505 and *R. brokii* D9, it unexpectedly shares the highest number of protein groups with *Nostoc* sp. PCC 7120, *Anabaena variabilis* ATCC 29413 and *N. punctiforme* PCC 73102 (data not shown). The species which shares the highest number of protein groups exclusively with NoAz is the facultative plant symbiont *N. punctiforme* (56 protein groups), while *C. raciborskii* and *R. brokii* only share a minor number of protein groups exclusive with NoAz (six and four groups, respectively). One explanation for this unexpected result is that *C. raciborskii* and *R. brokii* have lost genes, and that those which remain are most identical to genes in NoAz. A functional categorization of the protein groups shared between NoAz and the other nine cyanobacterial genomes compared shows that NoAz is most similar in this respect to *A. variabilis, Nostoc* sp. PCC 7120 and *N. punctiforme* (Figure S1). The categories mostly shared between NoAz and *N. punctiforme* are signal transduction mechanisms (T), cell membrane biogenesis (M) and carbohydrate and amino acid metabolism (G and E). All these categories, with the exception of signal transduction mechanisms, have an underrepresentation of pseudogenes in NoAz (Figure 4B). This is consistent with a selection pressure of such gene functions, and may indicate that these functional groups contain a large number of genes critical to symbiosis.

### Comparison to minimal essential gene sets

**Figure 6:**
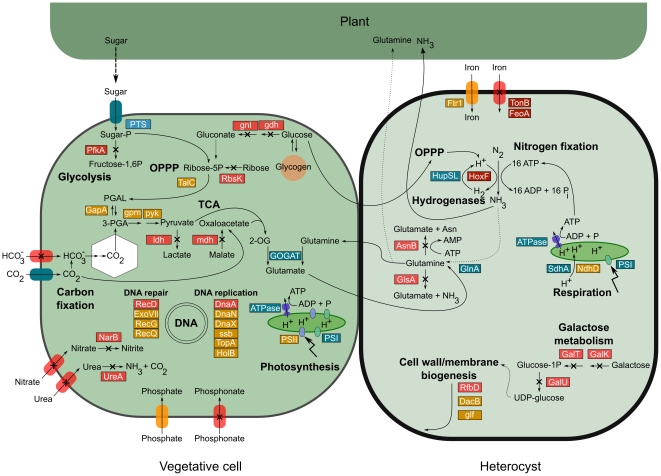
Schematic illustration of important metabolic and genetic information pathways in NoAz. The left cell represents a vegetative cell while the right a nitrogen-fixing heterocyst. Red color indicates pseudogenes lacking a functional counterpart in the NoAz genome. Orange indicates pseudogenes where a functional counterpart is present elsewhere in the genome. Fully functional gene(s) are illustrated (blue) only if their function is linked to other processes in the figure. The localization of pathways in vegetative cells or heterocysts is representative only for nitrogen fixation (heterocysts) and PSII activity (vegetative cells). Note that only a minor part of the nitrogen fixed in heterocysts is incorporated using the GS-GOGAT pathway and used for synthesis of amino acids, while most is exported to the plant as NH_3_. Sugar is provided by the plant in an as yet unknown form; putatively imported via the sugar phosphotransferase system (PTS). Function has been lost in the glycolytic pathway as the *pfkA* gene, encoding 6-phosphofructokinase, is a pseudogene and sugar metabolism in the *Azolla* cyanobiont probably proceeds via the Oxidative Pentose Phosphate Pathway (OPPP). Extensive loss of function is evident among genes involved in uptake and transport of nutrients and NoAz has lost the capacity to both import and metabolise alternative nitrogen sources. Table S3 shows detailed information on genes indicated in the figure and their closest homologs in other filamentous heterocystous cyanobacteria.

In order to understand the consequences of the eroding forces acting on the NoAz genome, comparative analyses were performed to the comprehensive minimal bacterial gene set [25], and the “core” and “shell” gene sets identified in cyanobacteria [26], encompassing 200 and 682 genes respectively. Like the genomes of the free-living *Nostoc/Anabaena* clade, the NoAz genome retains intact copies of most of the genes included in these two basic bacterial gene sets (Table S1, Table S2). Indeed, the maintenance of the *Azolla* symbiosis relies on key processes in NoAz specifically related to nitrogen fixation, cell differentiation and the vertical transmission process, all anchored in complex cellular developmental events (Figure 1). However, some crucial genes in the minimal bacterial gene set are non-functional or missing in NoAz (Table S1). The constant supply of nutrients from the plant host combined with a relaxed selection pressure may allow such eroding events to accumulate. These are related to glycolysis (*pfkA*, *gapA*, *pykA*, *gpmA*, *ldh*), to the basic replication machinery (the plasmid encoded *dnaN*, *dnaX* and *ssb*), the biosynthesis of cofactors (*nadR*) and the biosynthesis of nucleotides (*adk*). However, the NoAz genome contains a phosphoenolpyruvate-dependent sugar phosphotransferase system (PTS), a major carbohydrate transport system in bacteria [27], [28] which is lacking in most cyanobacterial genomes. Its presence in the NoAz genome indicates the capability of efficient import of carbohydrates (supplied by the plant) in the cyanobiont [16]. The fact that the gene encoding phosphofructokinase (*pfkA*), a key enzyme in the glycolytic pathway, is a pseudogene suggests that these imported carbohydrates are rather catabolized by the oxidative pentose phosphate pathway (OPPP). Compared to the cyanobacterial “core” and “shell” genes, defined by comparing 13 cyanobacterial genomes [26], additional loss of function are apparent in the genome of NoAz (Table S2). These are related to functions involved in translation (*lysS*), carbohydrate transport and metabolism (*melB*), co-enzyme transport and metabolism (*crtE*), defense mechanisms (*mdlB*), replication (*dnaA*) and amino acid transport and metabolism (*sdB*). Apart from glycolysis, impaired processes also relate to uptake of bicarbonate and phosphate, as well as import and utilization of alternative combined nitrogen sources (nitrate and urea). This suggests that nitrogen-fixation has been made an obligatory process in NoAz and a way to prevent any reassimilation of the nitrogen being released which is meant for the benefit of the plant. The restrained phosphate uptake may in turn explain the lack of polyphosphate granules in NoAz (data not shown). Besides the impairment in the ‘carbohydrate transport and metabolism’ function and in the uptake of bicarbonate noted above, NoAz apparently also suffers from a severely disrupted galactose metabolism (Figure 5B). The gene cluster involved is exclusive to the two symbiotically competent cyanobacteria (NoAz and *N. punctiforme*). The overall gene arrangement is shifted and multiple transposable elements are found in intergenic regions and even within the gene encoding UDP-galactopyranose mutase. Cyanobacterial host plants typically secrete carbohydrate rich mucilages that contains polymers (arabinose, glucose and galactose) to chemo-attract symbiotically competent cyanobacteria prior to plant colonization [7], [29]. As this capacity is superfluous in the perpetual *Azolla* symbiosis, gene clusters related to carbon uptake and metabolism may be subject to repeated deleterious mutations due to accelerated molecular evolution in the host-restricted environment. Also, a selective restriction in the supply of nutrients such as carbohydrates and phosphate, may be used to control and slow down the growth of NoAz, a phenotype also seen in all other cyanobacterial symbioses [7], [14]. Metabolic and informational processes negatively affected in NoAz are illustrated in Figure 6.

### Non-impaired gene sets and key functions

**Figure 7:**
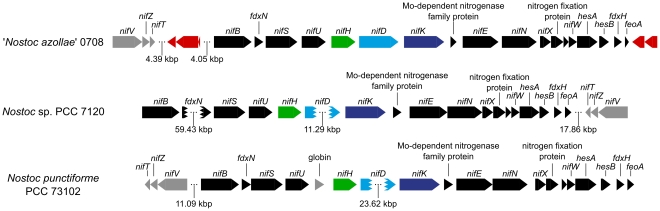
Illustration of genes related to N_2_-fixation, a highly conserved gene cluster in cyanobacteria. The structural genes for the nitrogenase enzyme (*nifHDK*) are highlighted in color for clarity. Also, genes which differ in terms of occurrence and/or organization are indicated in grey. The nitrogenase enzyme catalyzes the fixation of atmospheric dinitrogen gas. Transposases are indicated in red. Three dots indicate gaps and incision elements, with the length of the omitted sequence given.

In sharp contrast to the range of deleterious effects on the NoAz genome given above, is the number of intact genes related to symbiotically important physiological processes. A hallmark of all cyanobacterial-plant endosymbioses is the nutritional dependence on the cyanobionts by plant hosts. The total nitrogen demand of these often gigantic host plants (i.e. compared to the cyanobacteria) is fulfilled via highly efficient nitrogen fixation, which characterize all symbiotic cyanobacteria [7], [14]. Hence, as expected, the whole set of genes related to nitrogen fixation (the *nif* gene cluster) is intact, even though the *nif* operon is flanked by transposases (Figure 7). The 22 genes related to heterocyst formation [30], the cell type responsible for the nitrogen-fixing process in NoAz [31], are also present and intact. Moreover, the *nif* operon lacks DNA excision elements often found in heterocystous cyanobacteria [30]. Also lacking is the *patS* gene which encodes a small (13 or 17aa) suppressor of heterocyst development [30]. This gene includes the terminal “RGSGR” amino acid sequence signifying the functional motif of *patS* [32]. Notably, cyanobacterial *patS* mutants overproduce heterocysts, and a multi-heterocystous phenotype is a characteristic of NoAz (Fig. 1C; ∼20% heterocysts compared to 5–7% in free-living cyanobacteria; [14]). All genes necessary for the F-type ATPase are also present and intact which is not surprising considering the high ATP demand of the nitrogen fixation process. Likewise, genes essential for the function of photosystem I and II (PSI and PSII), the cytochrome b_6_/f, as well as a complete set of genes for synthesis of the light-harvesting biliproteins, are intact, although the copy number of *psbA* (encoding the D1 protein) is negatively affected with two intact genes and one pseudogene. The retainment of photosynthetic genes is unexpected, as the exposure to the low blue light in the cavity localized under the *Azolla* leaf/chloroplast ‘canopy’, may relax the normal photoautotrophic mode of cyanobacteria and rather promote a mixo- or heterotrophic life style dependent on carbon from the host plant [16]. However, a retainment of pigments is a signature for all cyanobacteria living in symbiosis with plants, including those living for years in the coralloid roots of cycads [7]. Genes involved in primary ammonia assimilation (*glnA* and GOGAT) are also intact, although most of the nitrogen fixed by NoAz is released as ammonia due to a ten-fold reduction in *glnA* transcript levels [33]. Additionally, our light and transmission electron microscopy analyses of NoAz reveal the presence of a range of subcellular structures (data not shown), which implies that all genes related to their synthesis must be present and functional. These include the photosynthetically active thylakoid membranes with phycobilisomes (containing light capturing pigments), numerous carboxysomes with the CO_2_ fixing enzyme ribulose-bis-phosphate carboxylase/oxygenase (RuBisCo), although a more than five-fold decrease in RuBisCo transcript levels have been shown earlier in the cyanobiont of *Azolla caroliniana* [33], a few cyanophycin granules and lipid droplets. Such data exemplify the numerous gene segments that are prerequisites in NoAz to fulfill its perpetual endosymbiotic role as a provider of combined nitrogen in the fast growing *Azolla*.

### Evolutionary aspects

While the process that led to the engulfment of a cyanobacterium to give rise to photosynthetic organisms is still unknown, it appears to have involved drastic erosions in and transfer of many genes from the cyanobacterium, resulting in the small genome size seen for chloroplasts of today (150–200 kb). Such “streamlining” of a genome is thus characterized by a loss of genetic material via gene deactivation and deletion. Given the large amount of pseudogenes in NoAz, it is evident that genes are currently being deactivated at a higher rate than they are being eliminated. Our results do not indicate that the NoAz genome has undergone recent genome shrinkage. The phylogenetic analysis (Figure 2, and Materials and Methods) suggest a scenario where NoAz has retained an ancestral genome size, and that larger changes in size has instead affected its closest relatives. It is possible that the ancestral genome size for the phenotypically complex cyanobacteria (Figure 2) was around 5 Mbp. Later, size reduction took place in the common ancestor to *C. raciborskii* and *R. brokii*, while genome expansion took place independently in the *Nostoc* spp./*Anabaena* clade and in the *Lyngbya*/*Arhtrospira*/*Trichodesmium* clade. Although it is not known whether *C. raciborskii* and *R. brokii* hold symbiotic competence, it is possible that the symbiotic association with *Azolla* is what has allowed NoAz to evade the genome shrinkage seen for its closest relatives. On the other hand, our results clearly reveal intriguing features signifying an eroding genome [9], [11] such as the abundance of pseudogenes, reflected in the diminished coding range, the large number of insertion elements and the A+T bias. Although the perpetual nature of the symbiosis and the loss of cyanobiont autonomy suggests a long-lasting strict co-evolution between the cyanobiont and its host, potentially extending over 140 million years, we argue, based on the genomic features shown here, that NoAz is in the early stage of genome erosion. Eventually, this process will favor genome deletions [9] which ultimately may cause NoAz to resemble a plant organelle (devoted to nitrogen fixation) more than a free-living organism. Notably, intermediates in organelle evolution are hitherto unknown and are postulated to require a vertical transmission process and an intracellular location of the symbiont [34]. We argue that the genomic features discovered here together with the highly sophisticated vertical transfer mechanism of the cyanobiont in the *Azolla* symbiosis, to safeguard propagation, do point in this direction although the cyanobiont resides extracellularly. In fact, the intracellular cyanobionts of the angiosperm *Gunnera* spp. are less intimate, characterized by facultative cyanobionts and horizontal transmission [7], [29]. Additionally, extracellular symbiotic bacteria of certain insects may undergo reductive evolution, as long as they are obligate and vertically transmitted [12], [13].

### Conclusion

*Azolla* is a globally widespread and fast growing symbiotic entity, most likely a consequence of its ability to use not only one, but two endosymbiotic cyanobacteria to gain the two most essential nutrients to sustain its great ecological fitness: one ‘ancient’ cyanobacterium to capture light energy and carbon (today chloroplasts), and one more recent endosymbiotic cyanobacterium to harvest dinitrogen gas. The latter is ensured by the evolution of a unique and safe propagation mechanism for vertical transfer of the nitrogen-fixing cyanobiont. The perpetual containment of the cyanobacterium within the plant body apparently allows this extracellular cyanobiont to undergo genome degradations of a kind hitherto unknown in any plant symbiosis and only seen in a few extracellular symbiotic bacteria [12], [13]. Moreover, the streamlining processes discovered in the genome of the *Azolla* cyanobiont is a reflection of its specific role as a continuous source of new nitrogen, and we speculate that it mimics the process that led to the evolution of the chloroplasts, also characterized by a series of consecutive genome erosions and selective gene retention events.

## Materials and Methods

### Strains and growth conditions

*Azolla filiculoides* was grown under greenhouse conditions with an 18/6 h (light/dark) diurnal cycle in tap water supplied with soil. The temperature was maintained at ca 30°C and the light varied according to natural daylight with addition of artificial light. Fronds of *A. filiculoides* were harvested and roots and decaying plant material was removed. The plants were then rinsed ten times with dH_2_O. Surface sterilization of the fronds was performed by stirring in 50% Clorox bleach for 15 min and subsequently rinsed 4×5 min in dH_2_O. The cyanobiont was isolated as previously described [35], with the modification of performing centrifugation in 40% Percoll up to 6 times. The cyanobiont purification procedure was examined and validated by PCR using cyanobacterial and bacterial 16S rRNA gene specific primers [36] followed by denaturing gradient gel electrophoresis [37]. High molecular weight genomic DNA was extracted from the cyanobacterial sample by enzymatic lysis and phenol/chloroform extraction (performed by BioServe, Beltsville, MD, USA).

### Structural analyses

Cyanobacteria were isolated from the plant as above and fixed in 2.5% glutaraldehyde before observing with an Axiovert 200 M inverted microscope (Zeiss). For transmission electron microscopy the cyanobacteria were prepared as previously described [38] and observed using ZEISS-EM 906 transmission electron microscope.

### Genome sequencing, assembly and annotation

The genome was sequenced using a combination of Sanger and 454 sequencing platforms. All general aspects of library construction and sequencing performed at the JGI can be found at http://www.jgi.doe.gov/. 454 pyrosequencing reads were assembled using the Newbler assembler version 1.1.02.15 (Roche). Large Newbler contigs were broken into overlapping fragments of 1000 bp and entered into assembly as pseudo-reads. The sequences were assigned quality scores based on Newbler consensus q-scores with modifications to account for overlap redundancy and to adjust inflated q-scores. A hybrid 454/Sanger assembly was made using the Arachne assembler. Together all sequence types provided 27.9× coverage (3.0× of Sanger data and 24.9× of pyrosequence) of the genome. Gene calling was performed at the Oak Ridge National Laboratory using the gene modeling program Prodigal [39]. Genome maps were plotted using DNAplotter [40]. Pseudogenes where annotated as such following the data cleaning protocol of the DOE Joint Genome Institute/Integrated Microbial Genomes (http://img.jgi.doe.gov/pub/doc/dataprep.html), which involves recognizing coding regions interrupted by more than one stop codon or frameshift, or being separated by another open reading frame, or corresponding to a truncated COG (or Pfam) less then 30% of the full-length COG. COG functional categories were assigned to genes and pseudogenes according to the DOE-JGI Standard operating procedure [41]. The distribution of pseudogenes in COG functional categories was tested using a Pearson's Chi-squared test with simulated p-value (based on 2,000 replicates).

### Genome comparisons

Gene orthology for the protein coding sequences in NoAz was assessed by comparing with the genomes of nine other cyanobacteria (Figure S1). Protein sequences corresponding to all annotated ORFs for the genomes were downloaded from NCBI (ftp://ftp.ncbi.nih.gov/genbank/genomes/Bacteria/) and JGI (http://img.jgi.doe.gov/). An all-by-all BLAST search (using NCBI blastp with the following parameters: -e 1e-05 -v 100000 -b 100000 -F ‘m S’), followed by Markov clustering into orthologous groups using OrthoMCL v.2.0-beta-6 [42], [43], were done to cluster protein sequences in orthologous groups. To classify proteins in the orthologous groups according to COG functional categories for all ten cyanobacterial genomes (of which *C. raciborskii* and *R. brokii* are not fully annotated) a local RPS-BLAST was performed as described in [41]. Origin of replication (*oriC*) regions were obtained from the Ori-Finder database [44].

### Phylogenetic analysis

The position of NoAz in the cyanobacterial phylogenetic tree was investigated using techniques similar to the Markov clustering followed by molecular systematics as outlined in [45]. Protein sequences corresponding to all annotated ORFs for 53 cyanobacterial genomes where downloaded from NCBI and JGI servers. An all-by-all BLAST search, followed by Markov clustering into orthologous groups were then done using OrthoMCL (see above). A set of single copy gene families present in all genomes were then selected and aligned using MUSCLE [46], and concatenated into a single alignment consisting of a total of 196,481 amino acid positions (476 genes/ORFs, see Table S4). The genome alignment was then analyzed under maximum likelihood as implemented in RAxML v.7.04 [47] using the WAG+GAMMA model and clade support was assessed using bootstrapping [48].

### Identification of insertion sequences

Remains of insertion sequences were identified by searching against the ISfinder [23] database using the genomic nucleotide sequence as query. All ISfinder repeats found using blastx (E-value cutoff <10) with the genome as query were collected, and RepeatScout v.1.0.5 [49] were used to collect repeats with lengths over 600 bp from the genome. Multi-copy genes with terminal inverted repeats in the NoAz genome were manually identified (since NoAz-specific insertion sequences were not in the ISfinder database). Blastn was then performed using whole genomes as queries against a local database of the collected sequences added to NCBI's non-redundant nucleotide database. Hits on the collected sequences with an E-value of <10^−5^ were considered as remains of insertion elements. Split reading frames were manually annotated into single insertions.

## Supporting Information

Figure S1 COG categories of orthologous protein groups shared between NoAz and nine related cyanobacteria. The highest number is shared with the heterocystous cyanobacteria in the Nostoc/Anabaena/Nodularia clade, particularly with Nostoc punctiforme PCC 73102, compared to those of the Cylindrospermopsis/Raphidiopsis and the three non-heterocystous representatives (Artm, Lyns and Trie). Abbreviations: Nosp  =  Nostoc punctiforme PCC 73102, Anav  =  Anabaena variabilis ATCC 29413, Noss  =  Nostoc sp. PCC 7120, Nods  =  Nodularia spumigena CCY9414, Cylr  =  Cylindrospermopsis raciborskii CS-505, Rapb  =  Raphidiopsis brokii D9, Artm  =  Arthrospira maxima CS-328, Trie  =  Trichodesmium erythraeum IMS101.
(1.92 MB TIF)

Table S1 Comparison of the minimal bacterial gene set to NoAz.
(0.06 MB XLS)

Table S2 Comparison of the cyanobacterial core and shell gene set to NoAz.
(0.19 MB XLS)

Table S3 List of pseudogenes in NoAz involved in processes shown in Figure 6.
(0.02 MB XLS)

Table S4 Genes used in the phylogenetic analysis of ‘Nostoc azollae’ 0708
(0.05 MB XLS)
