# Correction: Genome Erosion in a Nitrogen-Fixing Vertically Transmitted Endosymbiotic Multicellular Cyanobacterium

**DOI:** 10.1371/annotation/835c5766-5128-41c4-b636-adfe0c503103

**Published:** 2010-09-17

**Authors:** Liang Ran, John Larsson, Theoden Vigil-Stenman, Johan A. A. Nylander, Karolina Ininbergs, Wei-Wen Zheng, Alla Lapidus, Stephen Lowry, Robert Haselkorn, Birgitta Bergman

Table S2 is a duplicate of Table S3. Please view the correct Table S2 here: http://www.plosone.org/corrections/pone.0011486.s003.cn.xls

