# Supplementary figures and images for: Genome Erosion in a Nitrogen-Fixing Vertically Transmitted Endosymbiotic Multicellular Cyanobacterium
Source: PLoS One. 2010 Jul 8;5(7):e11486. doi: 10.1371/journal.pone.0011486 (PMC2900214; doi:10.1371/journal.pone.0011486)

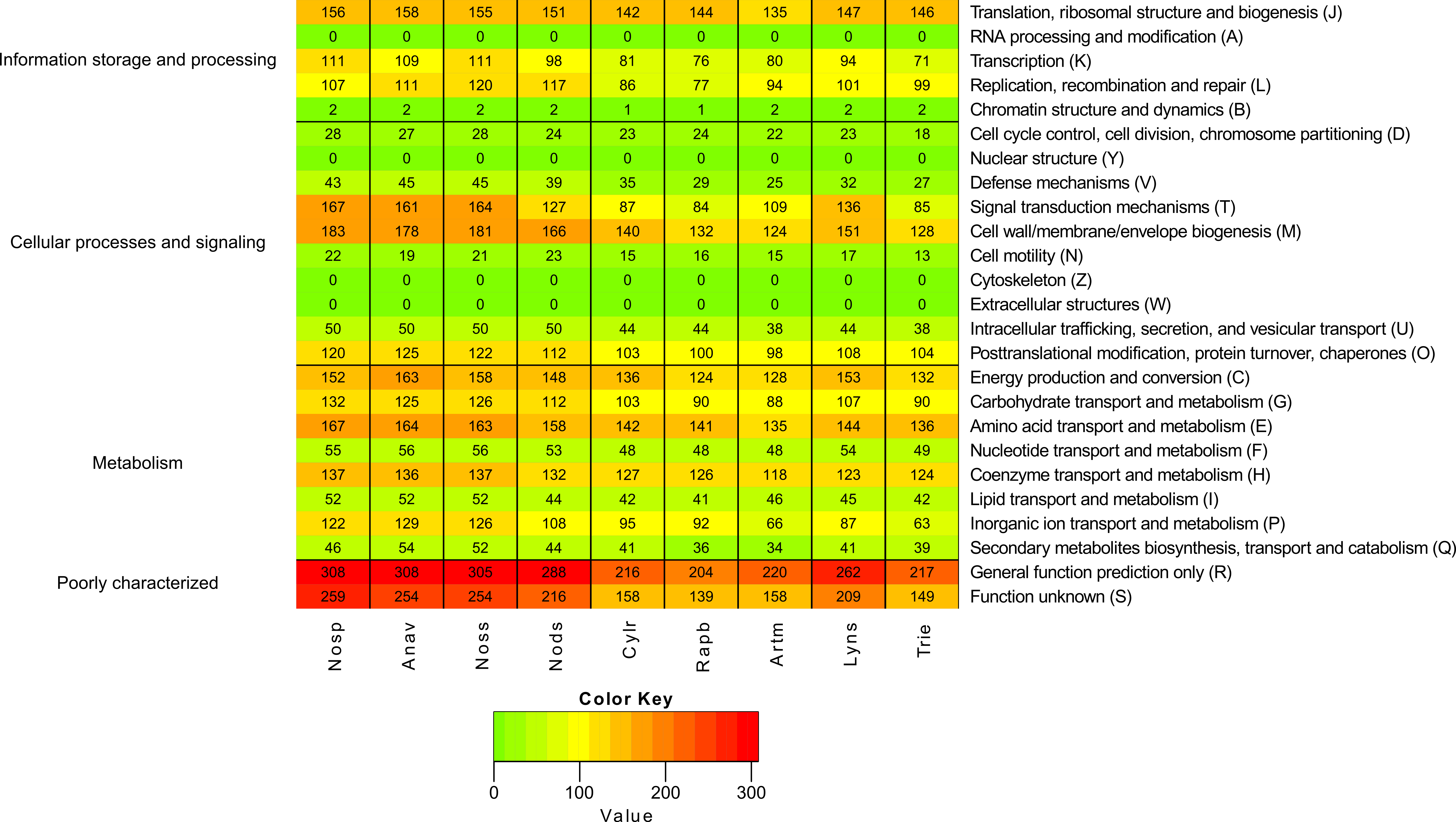

Supplement: Figure S1 — COG categories of orthologous protein groups shared between NoAz and nine related cyanobacteria. The highest number is shared with the heterocystous cyanobacteria in the Nostoc/Anabaena/Nodularia clade, particularly with Nostoc punctiforme PCC 73102, compared to those of the Cylindrospermopsis/Raphidiopsis and the three non-heterocystous representatives (Artm, Lyns and Trie). Abbreviations: Nosp = Nostoc punctiforme PCC 73102, Anav = Anabaena variabilis ATCC 29413, Noss = Nostoc sp. PCC 7120, Nods = Nodularia spumigena CCY9414, Cylr = Cylindrospermopsis raciborskii CS-505, Rapb = Raphidiopsis brokii D9, Artm = Arthrospira maxima CS-328, Trie = Trichodesmium erythraeum IMS101. (1.92 MB TIF) [file pone.0011486.s001.tif]
